# Bacterial vaginosis

**DOI:** 10.12688/f1000research.11417.1

**Published:** 2017-09-27

**Authors:** Phillip Hay

**Affiliations:** 1 Wandsworth Integrated Sexual Health, Courtyard Clinic, St. George’s Hospital, Blackshaw Road, London, SW17 0QT, UK; 2 Institute of Infection and Immunity, St. George’s, University of London, Cranmer Terrace, London, SW17 0RE, UK

**Keywords:** bacterial vaginosis, gardnerella, biofilms, sexually transmitted infections

## Abstract

Bacterial vaginosis is the most prevalent cause of abnormal vaginal discharge in women of childbearing age. It can have a major impact on quality of life and psychological wellbeing if frequently recurrent and strongly symptomatic. The use of molecular techniques to study the vaginal microbiome is increasing our understanding of the dynamic changes in flora that occur in health and disease. It might soon be possible to separate *Gardnerella *into different pathogenic and non-pathogenic species. Many groups are studying compounds that can disrupt the biofilm which is dominated by *Gardnerella *and *Atopobium vaginae*. Several studies in the last decade support the concept of bacterial vaginosis as a sexually transmitted infection.

## Introduction

Bacterial vaginosis (BV) is the most prevalent cause of abnormal vaginal discharge in women of childbearing age. Those most severely affected experience an offensive fishy-smelling discharge which recurs frequently, often around the time of menstruation. Others may have BV transiently and asymptomatically. It usually responds to treatment with antibiotics but can relapse rapidly, and reported rates of relapse are more than 50% within 3–6 months. There is a need for alternatives to antibiotics and to find a way to prevent relapse. Some recent studies imply that it is sexually transmitted, with more pathogenic strains of *Gardnerella* being identified. BV is a risk factor for adverse pregnancy outcomes, including second-trimester miscarriage, spontaneous preterm birth, and post-Caesarean section endometritis. It is also consistently associated with acquisition of sexually transmitted infections (STIs), including HIV.

## *Gardnerella*, biofilms and potential treatments

The description of a biofilm on the surface of the vaginal epithelium by Swidsinski and colleagues puts *Gardnerella*, as the dominant organism, once again at the centre of pathogenesis in BV ^1^. The role of *Gardnerella* as a sexually transmissible organism that initiates BV was elegantly discussed by Schwebke and colleagues in 2014 ^2^. Identifying differences in genes and phenotype between *Gardnerella* strains has been the focus of many studies. If non-pathological strains of *Gardnerella* exist, it could explain much of the epidemiology. Two strains of *Gardnerella*—one from a woman with BV and one without—were compared in 2010 ^3^. Differences were described in some genes and virulence factors such as adhesion, cytotoxicity and biofilm-forming capability. In a larger study, 17 isolates were studied and divided into genetically identified clades consistent with subspecies ^4^. Another study has confirmed four distinct subgroups of *Gardnerella*. Schellenberg and colleagues used sequencing of the chaperonin-60 universal target in a diverse group of 112 isolates ^5^. Sialidase activity, which is potentially an important mediator of pathogenesis through degradation of mucus, was identified in subgroups B and C but not A or D. Confusingly, the putative sialidase gene was present in some of the sialidase-negative clones. The authors conclude that it is likely that *Gardnerella* will be separated into four different species. A further study showed that strains differ markedly in their ability to degrade sialoglycans ^6^. It is possible that we may eventually be able to differentiate pathogenic from non-pathogenic *Gardnerella*, but it is also possible that there may just be a difference of degree of pathogenicity. There is concern about increasing levels of metronidazole resistance. I have recently seen several cases of clinically metronidazole-refractory BV. Specific clades have also been associated with metronidazole resistance. Schuyler and colleagues identified intrinsic metronidazole resistance in all of the studied strains from clades 3 and 4, but only 35% of clade 1 and 7% of clade 2: clades 1 and 3 were associated with BV and 2 and 4 were not ^7^. Interestingly, Alves and colleagues compared the ability of *Gardnerella* and 30 other BV-associated bacteria to form a biofilm, adhere to epithelial cells and induce cytotoxicity ^8^. *Gardnerella* was the most potent, once again supporting its central role in pathogenesis.

The demonstration of pockets of biofilm persisting after antibiotic treatment, and then recovering, has led researchers to study how to remove biofilm to achieve cure of BV ^9^. Whether relapse is due to this mechanism or to recolonisation from the gut or a partner has not been determined. Machado and colleagues extensively reviewed putative agents to potentially be used as adjunctive agents to antibiotics, including octenidine, boric acid, DNAses, retrocyclin, subtilosin, ploy-L-lysine, and lauramide arginine ethyl ester ^10^. At present, although some of them look promising, clinical evaluation has been limited. Prebiotics in the form of sugars used preferentially by lactobacilli and not by *Gardnerella* or *Candida* seem to be effective in restoring lactobacilli but might be needed in the long term to maintain a healthy vaginal flora ^11, 12^. Amphoteric tenside agents also look effective *in vitro*, and a clinical study of safety and efficacy has started ^13^.

The most recent licensed product for treating BV is dequalinium chloride. This is essentially an antiseptic agent. Repeated use, if not associated with toxicity, has to be preferable to a repeated course of antibiotics for those with frequently recurrent BV. The evidence for its efficacy was reviewed by Mendling and colleagues ^14^. This is mostly based on one large multicentre randomized controlled trial in which dequalinium chloride was equivalent to 2% clindamycin cream ^15^. It has some activity against *Candida* and so may be particularly useful for women who recurrently get both BV and *Candida*.

## Bacterial vaginosis as a sexually transmitted infection

Many studies over the last decade support the concept of BV as an STI, including the Australian study, which carefully evaluated any sexual activity as well as penile vaginal sex to define a virgin woman ^16^. Epidemiological evidence from several studies has established associations between BV and increased numbers of sexual partners, inconsistent condom use and young age at sexual debut. Zozaya and colleagues reported that sexual exchange of BV-associated bacterial taxa between heterosexual couples is common ^17^. Molecular sequencing has shown that males carry BV-associated bacteria in the sub-preputial space and distal urethra ^18, 19^, and in one of these, certain bacteria were associated with non-gonococcal urethritis ^18^. Additionally, in another study, BV-associated bacteria were more prevalent in male partners of females with BV than without ^20^, and there was concordance of specific BV-associated bacteria and of specific subgroups of *Gardnerella* in heterosexual couples ^21^.

Historically, several studies established that there is a high rate of concordance for BV within couples of women who have sex with women. More recently, the concept of sexual transmission between women was supported by findings that incident BV is associated with a new female partner, a female partner with BV and receptive oral sex in two prospective cohorts ^22, 23^.

However, if BV is sexually transmitted, we do not know whether the condition results from transmission of a single agent. Thus, Muzny and Schwebke propose a model with *Gardnerella* as the ‘keystone’ organism for the development of BV, with acquisition of both *Gardnerella* and additional anaerobes being sexual ^24^. On the other hand, Swidsinski and colleagues described biofilm in the urine of male partners of women with BV and described acquisition of BV in a woman receiving semen including biofilm for artificial insemination ^25, 26^, implying that it is transmitted as a polymicrobial consortium. The early studies by Gardner, Dukes and colleagues support the latter, as they were more successful in transmitting BV with fresh vaginal fluid than a pure growth of *Gardnerella* ^27^.

*Gardnerella* is found in the gut as well as in the vagina and penis. In a thorough review, Kenyon and Osbak discuss the interchange of bacteria from a woman’s vagina, oral and rectal microbiomes, and those of her partner and the partner’s other sexual contacts ^28^. This implies a role for BV-associated organisms in the gut to provide a source of recolonisation. Even amongst men who have sex with men (MSM), BV-associated organisms are prevalent in the rectum. A study of 107 MSM in Belfast found *Gardnerella* in 83.2% of rectal samples and *Mycoplasma hominis* in 24.3% ^29^. If sexual contact leads to initial colonisation with BV, it is remarkably persistent thereafter in many women. Perhaps the best evidence that male partners contribute to BV was the approximately 50% reduction in BV in women whose partners were randomly assigned to circumcision in an HIV prevention study ^30^. Against the STI argument, the rates of persistent/recurrent STIs such as HSV-2 infection increase with age whereas BV rates were the same through ages 20–49 in a large study from the USA ^31^.

## Sex hormones and microbiota

A study of 682 women looked at the correlation between contraceptive method and vaginal microbiome ^32^. They found that women using combined oral contraception (COC) (odds ratio (OR) 0.29) and depot medroxyprogesterone acetate (DMPA) (OR 0.34) had significantly reduced rates of colonisation with BV-associated bacteria compared with those using condoms. Those with COC also were more likely to be colonised with H _2_O _2_-producing lactobacilli (OR 1.94). Women using an intrauterine system had greater rates of BV-associated bacteria, but this was not statistically significant. This supports the conclusion from an earlier study in which hormonal stimulation for *in vitro* fertilization was associated with a decreased rate of BV ^33^. Combined hormonal contraception taken for 3 months continuously is a strategy that needs to be studied for reducing BV by providing both increased oestrogen levels and reduced frequency of menstruation. This is in keeping with two systematic reviews from 2013 which identified the association between use of specific hormonal contraception and reduced risk of BV ^34, 35^.

Several studies of the vaginal microbiome have been published in recent years. Understanding what is normal vaginal flora is clearly relevant to understanding dysbiosis. A large cross-sectional study of 396 healthy reproductive-age women used culture-independent approaches to classify vaginal bacteria composition profiles into five community state types ^36^. Four relatively stable lactobacillus-dominated community state types were identified in 73% of women by using cultivation-independent methods based on the analysis of 16S ribosomal RNA (rRNA) gene sequences. A fifth corresponded more with dysbiosis and BV. However, many of these bacteria produce lactic acid and some of these microbiomes were not associated with symptoms ^37^. A full discussion of these developments appeared recently in a whole journal issue devoted to the vaginal microbiome with a thorough editorial written by Crucitti ^38^. ****

## Conclusions

Molecular techniques are giving us new insights into BV. Is there a pathogenic type of *Gardnerella* that is sexually transmitted and can be distinguished from benign planktonic strains? Or is BV merely sexually associated, such that *Gardnerella* carriage and exposure are almost ubiquitous but something happens with a new sexual partner that allows BV to develop? Further studies of the microbiome need to define the apparent normal flora in some women that is not dominated by lactobacilli. We might be better able to develop probiotic therapies if we can better understand the relationship between host and vaginal flora ^39^. We desperately need better ways of preventing recurrence that do not involve repeated courses of antibiotic. There are several candidate therapies which need to be taken into clinical studies.
